# Tensile strength assay comparing the resistance between two different autologous platelet concentrates (leucocyte-platelet rich fibrin versus advanced-platelet rich fibrin): a pilot study

**DOI:** 10.1186/s40729-020-00284-w

**Published:** 2021-01-15

**Authors:** Martim de Almeida Nóbrega Correia Pascoal, Nuno Bernardo Malta dos Santos, António Manuel Godinho Completo, Gustavo Vicentis de Oliveira Fernandes

**Affiliations:** 1 Integrated Master in Dental Medicine, Faculty of Dental Medicine, Universidade Católica Portuguesa, Viseu, Portugal; 2 Periodontics Department, Center for Interdisciplinary Research in Health (CIIS), Faculty of Dental Medicine, Universidade Católica Portuguesa, Viseu, Portugal; 3 TEMA, Universidade de Aveiro, Aveiro, Portugal; 4 Implantology and Biomaterials Department, Faculty of Dental Medicine, Universidade Católica Portuguesa, Quinta da Alagoa Ave., 225 – 1 DT, 3500-606 Viseu, Portugal

**Keywords:** Fibrin, Rupture, Platelet, Tensile strength, Resistance

## Abstract

**Background:**

Since the leucocyte-platelet rich fibrin (L-PRF) was published in 2001, many studies have been developed, analyzing its properties, and also verifying new possibilities to improve it. Thereby, it emerges the advanced-platelet rich fibrin (A-PRF) with a protocol that optimizes the properties obtained by the L-PRF. Nonetheless, there is a gap in the literature to landmark the evolutive process concerning the mechanical properties in specific the resistance to tensile strength which consequently may influence the time for membrane degradation. Thus, this study had the goal to compare the resistance to the traction of membranes produced with the original L-PRF and A-PRF protocols, being the first to this direct comparison.

**Findings:**

The harvest of blood from a healthy single person, with no history of anticoagulant usage. We performed the protocols described in the literature, within a total of 13 membranes produced for each protocol (*n* = 26). Afterward, the membranes were prepared and submitted to a traction test assessing the maximal and the average traction achieved for each membrane. The data were analyzed statistically using the unpaired *t* test. Regarding average traction, A-PRF obtained a value of 0.0288 N mm^−2^ and L-PRF 0.0192 N mm^−2^ (*p* < 0.05 using unpaired *t* test). For maximal traction, A-PRF obtained 0.0752 N mm^−2^ and L-PRF 0.0425 N mm^−2^ (*p* < 0.05 using unpaired *t* test).

**Conclusion:**

With this study, it was possible to conclude that indeed A-PRF has a significative higher maximal traction score and higher average traction compared to L-PRF, indicating that it had a higher resistance when two opposing forces are applied.

## Introduction

The most frequently used biomaterial are the ones that come from autologous sources, which is still and considered to be the “gold standard” due to all its properties, including induction, conduction, and genesis, besides preventing the risk of infection [1]. These biomaterials can be produced from hard or soft tissue (e.g., bone and connective tissue, respectively), or the blood. In the past years, autologous blood concentrates have been traditionally used in transfusions aiding in the control of hemorrhage caused by severe thrombocytopenia, often associated with multiple blood illnesses or because of blood loss during long surgeries [2].

Historically, there are three generations of autologous platelet concentrates (APCs). The first was mainly represented by platelet-rich plasma (PRP) that was produced with the introduction of an anticoagulant (sodium citrate, EDTA) and other compounds (calcium chloride, bovine thrombin) in the collection tubes and it required two centrifugations. This product has shown to be useful on certain occasions as the literature has shown [3].

Other APCs are described as fibrin glues (used to seal wounds and promote healing) [3], the platelet gel [4]. And plasma rich in growth factors (PRGF) [5] which required not only the addition of an anticoagulant but also of calcium chloride, to activate platelets resulting in the release of growth factors [6]. However, the PRGF protocol proved to be problematic due to its lack of reproducibility and leading to a greater possibility of obtaining undesirable tissue response [5, 7].

The second generation was introduced in 2001 with a smaller cost of production, easier handling, and better success rates in clinical cases [1, 8, 9], represented primarily by leucocyte-platelet rich fibrin (L-PRF®), which were able to create a superior scaffold [10]. This material was produced in a dry tube with no added compounds within it. Biochemical analysis of the PRF indicates that this biomaterial consists of the presence of cytokines, glycan chains, and structural glycoproteins involved in the fibrin network that was slowly polymerized. These components have demonstrated synergistic healing processes [1, 8, 11], mainly due to the releasing of cytokines [1, 8–10, 12] released from the three-dimensional fibrin matrix which is continuously reabsorbed, inducing a greater response in the healing process [13].

Afterward, researchers sought to produce an even better membrane with greater biological properties, using the low-speed centrifugation concept (LSCC). The evidence that protocols with reduction of the centrifugation force allow a greater and better distribution of cells of interest for the effectiveness of PRF in tissue regeneration, which resulted in the production of Advanced-Platelet Rich Fibrin (A-PRF®) [12, 14]. This protocol was conceived to optimize the properties of the clot produced by the L-PRF technique to achieve a more appropriate scaffold with an even population of cells [7] and also containing greater numbers of white blood cells (neutrophils, macrophages, B and T lymphocytes) [13, 15]. Since then, PRF has been heavily applied in the dentistry and medical fields [7, 12, 16].

The third generation proposed a modification, introducing concentrated growth factors (CGF) [17, 18] changing the centrifugation speed, from 2400 rpm to 3000 rpm, and the centrifugation periods. Characterized by containing abundant growth factors in its rigid fibrin [19], yielding results in speeding up the proliferation and differentiation of cells [20].

However, the preparation protocols of A-PRF and CGF are similar and share the same principle in clot formation. They are not distinguishable either macro or microscopically, and to the present date, there have not been found any significant differences between them [19].

Three-dimensional scaffolds (2nd and 3rd generations) allow a continuous release of cytokines and growth factors enhancing mainly the first period of tissue repairs, such as TGF (transforming growth factors), PDGF (platelet-derived growth factors), VEGF (vascular endothelial growth factors), IGF (insulin-like growth factors), and many others, enhancing the healing process [3, 15], throughout almost 10 days [7] regulating the inflammation and reducing the risk of infection [20]. In comparison, PRP (1st generation) is completely dissolved in 3 days releasing its growth factors in the first hours [1, 21].

In this evolutive perspective, L-PRF and A-PRF have been considered to have improved mechanical properties [1] but there is no direct comparison of the L-PRF and A-PRF properties. Khorshidi et al. [22] tested the mechanical properties of early L-PRF versus PRGF/Endoret membranes. Another study analyzed the addition of silver nanoparticles into L-PRF in a way to improve its mechanical characteristics [23]. Isobe et al. [19] developed a comparison between A-PRF and CGF evaluating the mechanical parameters and degradability. Both groups were almost identical. Nonetheless, the highlight has been given to A-PRF clots who display a higher concentration of growth factors, inducing a more significant effect on angiogenesis, and its characteristics will surely deliver a different resistance to the membrane [24].

Thus, there is a gap in the literature to landmark the evolutive process concerning the mechanical properties in specific the resistance to tensile strength which consequently may influence the time for membrane degradation. Furthermore, there is no direct comparison in the literature of these two protocols (L-PRF and A-PRF) and how these products behave mechanically, this research may be a tool to further extend the knowledge on how these materials will interact in a surgical wound and help in clarifying what results should the clinician expect to achieve.

Therefore, this research aimed to evaluate and compare the value of mechanical resistance to the tension of PRF membranes produced with different protocols (L-PRF and A-PRF). The hypothesis of this study was to verify the membrane with better tensile properties and be able to suggest a product more able to withstand the stress when applied in surgeries.

## Materials and methods

### Blood collection and membrane preparation

**Fig. 1:**
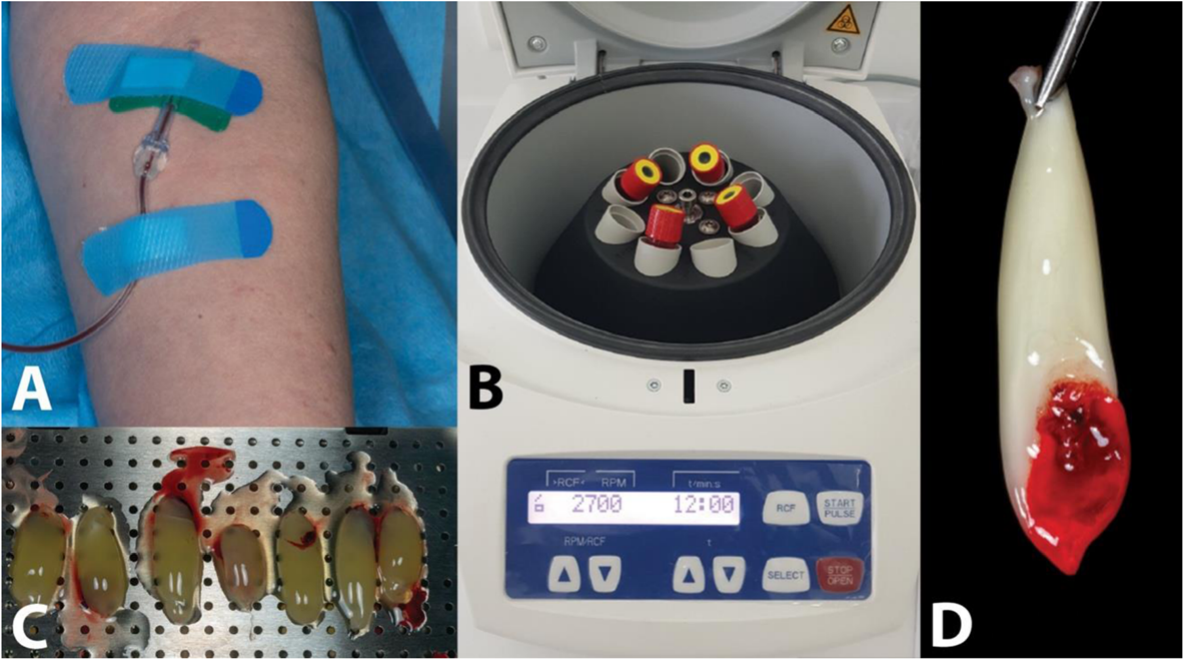
PRF membrane production protocol. **a** Blood harvesting. **b** Blood centrifugation. **c** Clots collected from the centrifuged tubes and placed in the Xpression box kit. **d** Final PRF membrane obtained

The design of this study and its consent forms for all procedures performed followed the Helsinki Declaration of 1975 as revised in 2013, and the study started after approval by the Ethics Committee (number 522020). Then, the blood was collected from a single healthy person (M.A.N.C.P.) in different days avoiding variations, under restricted food starting 1 day prior to the procedure, with no history of anticoagulant usage or any disease, into 9-mL sterile glass-coated plastic tubes, red top blood collection tubes (Intralock©, USA) (Fig. 1a), under standard ambient conditions at 20 ± 2 °C. The study was performed in collaboration with the Centre for Mechanical Technology and Automation (TEMA) of the University of Aveiro (Portugal).

L-PRF membranes were prepared according to the original technique with centrifugation at 2700 revolutions per minute (rpm), 408 g, for 12 min with the IntraSpin™ centrifugation device (33° rotor angulation, 50 mm radius at the middle of the tube, 80 mm at the maximum, and 40 mm maximum, and 40 mm at the minimum) (Intra-Lock, Boca Raton, FL, USA) [23] (Fig. 1b). For the A-PRF membrane preparation, both the centrifugation time and speed are different, following the original values for this technique 1500 rpm (126 g) for 14 min [12, 24].

After 12 min of centrifuging for L-PRF and 14 min for A-PRF, beyond the membranes rested inside the box for 20 min before performing procedures [25], the clots are ready (13 membranes for each group, totaling *n* = 26). Thus, the fibrin clots were taken out of the tubes and separated from the red blood cells. Following membrane preparation, fibrin clots were placed in the Xpression box (IntraLock©) for gentle compression by gravity and slightly pressing until to close completely the metal cover (Fig. 1c), following the recommendation of the manufacturer. Five minutes later, the L-PRF and A-PRF membranes are ready for use (Fig. 1d).

### Tensile assay

**Fig. 2:**
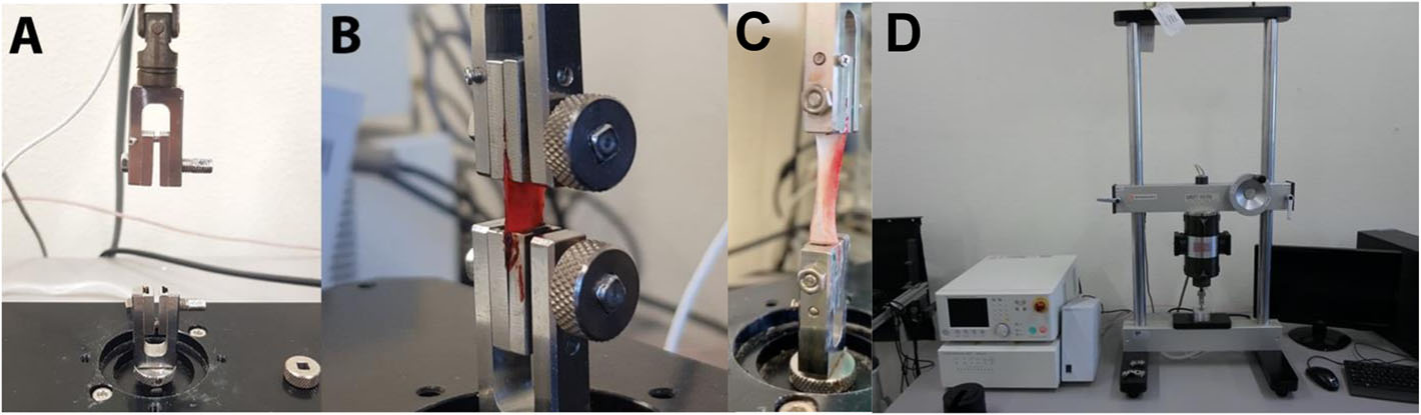
Mechanical traction assay. **a** Equipment where all tests were performed. **b** Execution of the test in the L-PRF group. **c** In A-PRF group. **d** Equipment used to perform all mechanical tests, Shimadzu MMT-101 N (Shimadzu Corporation; Japan)

Before the traction test, the membranes were standardized and measured using a WHO Periodontal probe and cut in a rectangular shape in which the short ends measured 5 mm of length and 1 mm of height each, only one per membrane. The tensile test was performed using a universal testing machine (Shimadzu MMT-101 N equipment, Shimadzu Corporation, Japan) [22, 26] (Fig. 2a), where the PRF membranes were conducted through a surgical tweezer, to put both extremities of the membranes fixed in the tensile force apparatus. By applying divergent forces (1 mm spacing between the claws of the equipment), the maximum traction was measured in 13 membranes for each protocol (*n* = 26) (Fig. 2b), until rupture [27]. The maximum value for traction using this equipment is set to 12 mm.

The equipment worked with the same movements for all membranes. Likewise, the position of the equipment was similar according to the time of analysis, during the traction of the PRF. Therefore, the variable time was not considered for the study and it was not included. But, the variable resistance to tension (start of traction and end, rupture) were evaluated.

### Data and statistical analysis

The force applied to the membrane per area of the section in the equipment’s claws (N mm^−2^), and the traction of the membrane (percentage of deformation in comparison to the initial spacing between the claws, 1 mm) were plotted for each membrane. With all the information needed, a graph was constructed to determine the maximum force that was applied until the membrane ruptured, giving us the maximum tensile strength.

Data was collected and transferred to Microsoft Excel (Microsoft©) and GraphPad Prism 7.0 where all statistical analysis was performed. All normality tests evaluated the data obtained. Values are presented as mean ± SEM in the figure legends. Statistical comparisons included a two-sided unpaired *t* test. A significant level of significance was obtained if the *p* value was ≤ 0.05. Moreover, it was verified the normal distribution of the means found (Normal Q-Q analysis).

## Results

**Fig. 3:**
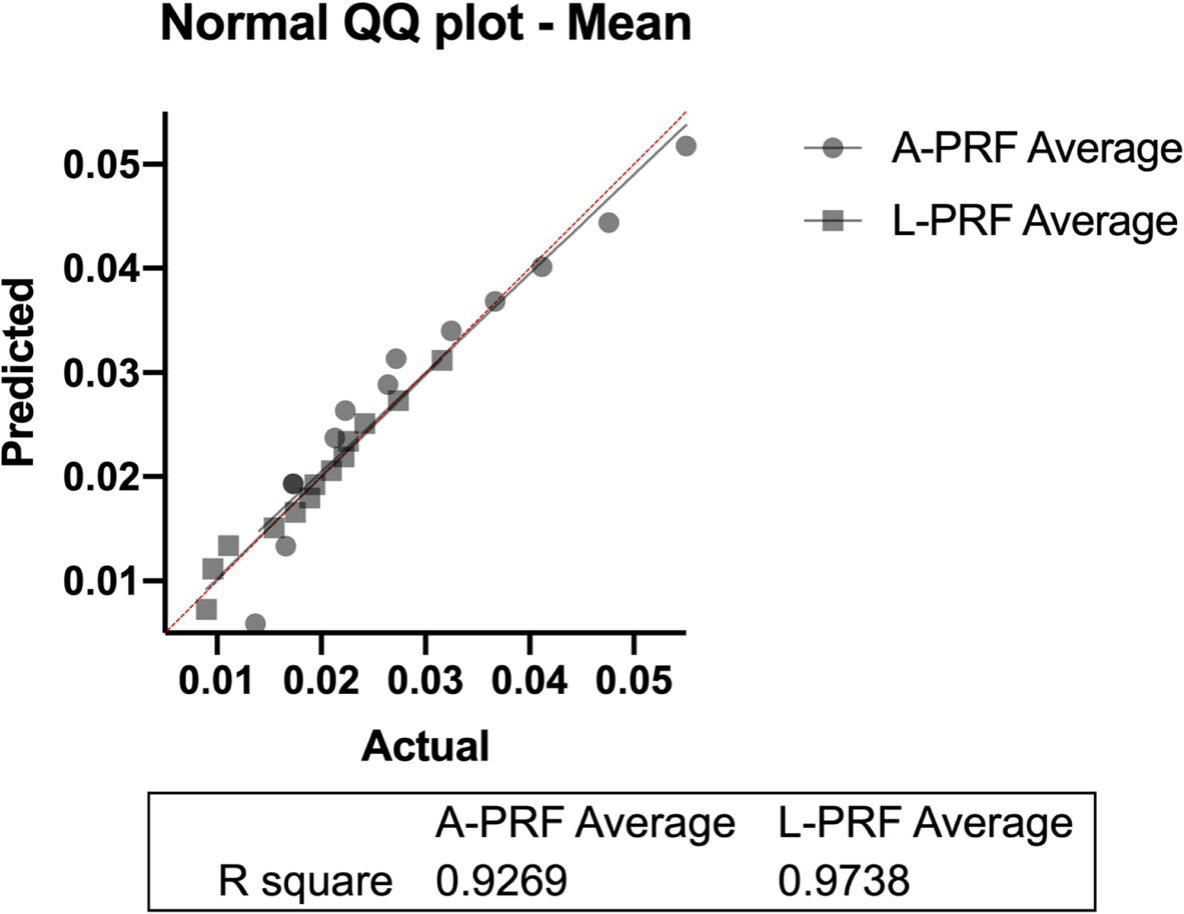
Normal Q-Q plot. Correlation of the data with the normal distribution (line)

The traction evaluation was based on the quantification of the average traction obtained for each membrane tested and the maximum value detected upon the rupture of each membrane. A curve of the values obtained was registered to observe a possible correlation and comparison between the groups. This proved important not only to discover the maximum resistance of the membranes but also to understand if it would represent an actual statistically different average resistance. All data were analyzed and they were according to the normality (Table 1) what can be verified positive correlation with the normal distribution obtained (Fig. 3).

**Table 1:** Test for normal distribution

Number of membranes	L-PRF	A-PRF
Normality tests	13	13
***D’Agostino and Pearson test***		
*P* value	0.9763	0.4109
Passed normality test (alpha = 0.05)?	Yes	Yes

**Fig. 4:**
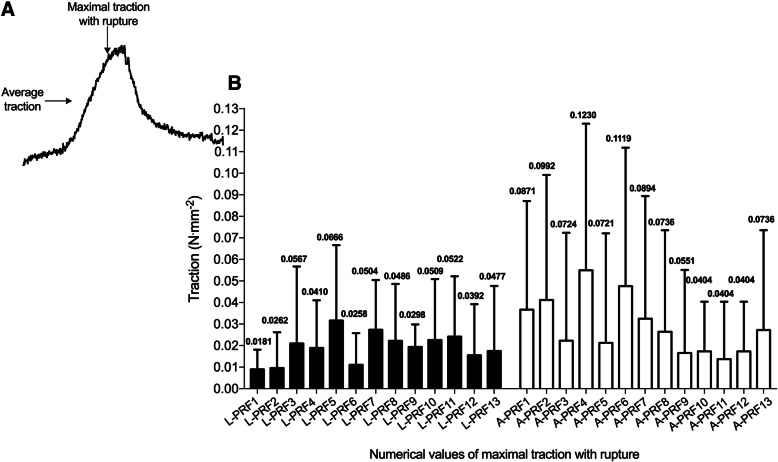
Average and maximal traction values. **a** Representative traction profile with maximal traction with rupture of membrane and average traction identified by arrows. **b** Individual values of each membrane tested for each protocol with average values of traction, and maximal value for traction measured

From the traction evaluation of 13 L-PRF and 13 A-PRF membranes, it was found that there was a significant statistical difference in the maximum traction with rupture and the average traction between the L-PRF and A-PRF protocols (Fig. 4a, b). The traction test results had some variability within and between groups. All detailed data were demonstrated in Table 2.

**Table 2:** Descriptive statistics of the traction data obtained

Average	Maximal limit	
	L-PRF	A-PRF
**Minimum**	0.009000	0.01370
**Maximum**	0.03160	0.05500
**Range**	0.02260	0.04130
**Geometric mean**	0.01802	0.02637
**95% CI of median**		
Actual confidence level	97.75%	97.75%
Lower confidence limit	0.01110	0.01730
Upper confidence limit	0.02420	0.04120
**Mean, SD, and SE**		
Mean	0.01923	0.02885
Std. deviation (SD)	0.006760	0.01297
Std. error of mean (SE)	0.001875	0.003597
**95% CI of mean**		
Lower	0.01515	0.02102
Upper	0.02332	0.03669

**Fig. 5:**
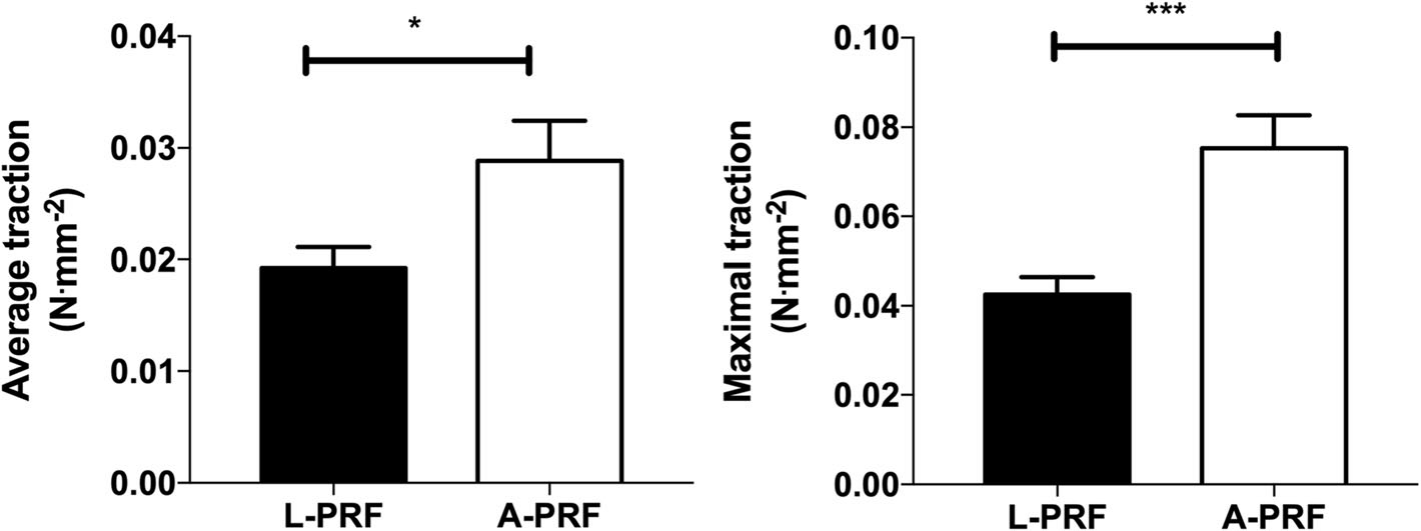
Average and maximal traction values. **a** Average traction difference between L-PRF and A-PRF protocol (*p < 0.05 using unpaired t test n = 13). **b** Maximal traction difference between L-PRF and A-PRF protocol (***p < 0.001 using unpaired t test *n* = 13)

In reference to the average traction, A-PRF obtained a value of 0.0288 N mm^−2^ and L-PRF 0.0192 N mm^−2^ (*p* ≤ 0.05 using unpaired *t* test) and for maximal traction, A-PRF obtained 0.0752 N mm^−2^ and L-PRF 0.0425 N mm^−2^ (*p* < 0.001 using unpaired *t* test) (Fig. 5a, b).

## Discussion

This study intended to fill a lack of scientific knowledge about the APC membranes’ mechanical properties, evaluating specifically the resistance to tension until rupture between L-PRF and A-PRF. It is an important question, especially regarding the degradation time which may be different from a tougher membrane. If the biomaterial is rapidly reabsorbed, it may lead to insufficient tissue regeneration [28]. Also, harvesting the blood of only one participant avoided any interference or bias.

In Medicine, these membranes can be applied in refractory leg ulcers associated or not with osteomyelitis [29], improving wound healing and closure. In Dentistry, more specifically in periodontal surgery, questions have emerged about the use of PRF membranes with favorable results. Studies have suggested it as a possible substitute for a connective tissue graft, which is still considered the “gold standard” for soft tissue surgery [16, 30]. In oral surgery, favorable results have been found for the insertion of membranes inside of fresh socket after extraction or using it in the treatment for bone lesions [31]. Furthermore, the use of a PRF membrane avoids a donor site which greatly decreases the postoperative discomfort [30].

An article evaluated the performance of APC associated with albumin [32] which could represent a possible improvement in its framework, modulating the fibrin network ultrastructure and permeability. Also, the material produced in this study showed to have higher biocompatibility, and possibly more durable with a greater thickness and resistance [32, 33]. However, the levels of released cytokines and growth factors were similar to those found for PRF; also, its degradation period is compatible with PRF extending to almost 10 days [1, 7]. Being more difficult and expensive to produce this technique proves to be inferior to a PRF membrane.

This biodegradable material, APC, suffers degradation after its insertion in the surgical site, becoming necessary to know its resistance. Furthermore, freezing the membranes could be an alternative methodology to improve the characteristics of APC, at temperatures of −20 °C, and thawing at +4 °C, which may help to decrease the rapid degradation, becoming a better biomaterial for clinical application [28]. Although it may be hard to control the correct temperature on the day of application, the use of a freezer with the desired settings may prove beneficial.

Another methodology was developed and deserves to be highlighted. It is known as the low-speed centrifugation concept (LSCC) which is used to produce A-PRF membranes. This concept is suggested to be the factor that greatly increases the resistance of the membranes produced with this protocol. This technique diminishes the cell pull-down by the g forces applied in the centrifugation, increasing the number of cells within the top layer of the fibrin matrix. This surely modifies the A-PRF’s properties compared to L-PRF, which suffers much higher forces during the centrifugation, concentrating almost all cellular content at the bottom of the clot [24].

Controversially, although other techniques [28, 32] exist, the simplest way is to follow the protocols strictly using the correct centrifugation settings to obtain the correct membrane. Thus, the results obtained for L-PRF in this study (average of 0.02260 MPa) is in perfect agreement with the resistance of L-PRF published by Khorshidi et al. [22] (0.20 ± 0.06 MPa), but strangely, Ravi and Santhanakrishnan (2020) [26] found an extremely higher value for L-PRF (290.076 ± 5.68 MPa). Nonetheless, the same authors also found extremely high values for A-PRF (362.565 ± 5.15 MPa), differently than obtained in this study (average of 0.04130 MPa). However, this study may state that A-PRF had a higher resistance to traction than L-PRF similar to published by Ravi and Santhanakrishnan (2020) [26]; therefore, in this study, it was observed almost twice more in the average of resistance, with an extremely significant statistical difference. Concerning average traction, A-PRF also achieved a significant statistical difference compared to L-PRF. This fact is due to a looser structure with more interfibrous space with a lower crosslink and greater elasticity, beyond the better distribution of the content throughout the fibrin after using the LSCC, which allowed a greater presence of neutrophil cells and platelets in the PRF [12, 34, 35].

It was noticed that in the A-PRF group some membranes scored similarly, regarding the maximal traction. However, the average traction was different, indicating that the structure of each membrane was slightly different, even with the same donator and time of the blood collection. In the L-PRF protocol, the same happened but with little difference between the values. This indicates that there is a large variation to be expected when producing APC membranes, and one may not achieve the highest traction possible. Regarding the experimental time, it was observed for L-PRF the mean of 72.33 s (SD ± 41.84 s) with a maximum time of 180.40 s; and for A-PRF, the mean of 74.48 s (SD ± 43.15 s) with maximum period of 180.50 s. For period analyzed to achieve rupture, for L-PRF group was found the average of 74.73 s (minimum 34.60 s and maximum 130.40 s); while for A = PRF group, the average was 132.24 s, almost twice the L-PRF group, with minimum 81.50 s and maximum 178.50 s.

It was known that A-PRF had a higher concentration of growth factors within its fibrin matrix, increasing the tissue regeneration rate when applied in a surgical wound [12]. This fact allied to the higher maximal traction and average traction appears to make it a more suitable material for regeneration than L-PRF. Indeed, the LSCC has produced membranes with a more even distribution of cells throughout the fibrin clot and a more mechanically resistant membrane. This reveals that, when applied in multiple situations, A-PRF may be more effective.

Regarding the only one individual included in this study, this strategy was applied to avoid any type of bias. The literature [36–38] has reported platelet alterations associated with many systemic conditions and the use of drugs, which was observed and controlled in this study.

## Conclusion

Through this pilot study, it was possible to conclude that there was significantly higher resistance to traction in membranes produced with the A-PRF compared to the L-PRF protocol, suggesting more handleability when applied clinically, such as in surgeries, resisting receiving a suture. Nevertheless, it is necessary that more studies with major samples and distinguishing gender and age.

## Data Availability

All data are included in the article.

## Abbreviations

APC: Autologous platelet concentrate
A-PRF: Advanced-platelet-rich fibrin
CGF: Concentrated growth factors
CI: Confidence interval
EDTA: Ethylenediaminetetraacetic acid
IGF: Insulin-like growth factors
L-PRF: Leucocyte- and platelet-rich fibrin
LSCC: Low-speed centrifugation concept
PDGF: Platelet-derived growth factors
PRF: Platelet-rich fibrin
PRGF: Platelet-rich growth factors
PRP: Platelet-rich plasma
SD: Standard deviation
SE: Standard error
SEM: Standard error of the mean
TGF: Transforming growth factors
VEGF: Vascular endothelial growth factors
WHO: World Health Organization
